# Etomidate as an anesthetic in *Colossoma macropomum*: Behavioral and electrophysiological data complement each other as a tool to assess anesthetic safety

**DOI:** 10.1371/journal.pone.0305093

**Published:** 2024-08-06

**Authors:** Thaysa de Sousa Reis, Daniella Bastos de Araújo, Clarissa Araújo da Paz, Rodrigo Gonçalves Santos, Anara de Sousa Barbosa, Luana Vasconcelos de Souza, Yris da Silva Deiga, Vera Louzeiro de Oliveira Garcia, Gabriela Brito Barbosa, Lucas Lima da Rocha, Moisés Hamoy

**Affiliations:** Laboratory of Pharmacology and Toxicology of Natural Products, Biological Science Institute, Federal University of Pará, Belém, PA, Brazil; Rajasthan University of Veterinary and Animal Science, INDIA

## Abstract

The use of anesthetic agents in the management of fish in fish farming or ornamental fish breeding aims to minimize stress and promote animal welfare. Therefore, this study aims to investigate behavioral, electrocardiographic, and ventilatory characteristics of tambaquis exposed to anesthetic baths with etomidate. The study was conducted with juvenile tambaquis (27.38 ± 3.5g) n = 99, at etomidate concentrations of 2–4 mg.L ^-1^, analyzing induction and anesthetic recovery behavior (experiment I), electrocardiogram (experiment II), and opercular movement (experiment III). Fish exposed to high concentrations of etomidate reached the stage of general anesthesia faster, however, the recovery time was longer, characterizing a dose-dependent relationship. Cardiorespiratory analyzes demonstrated a reduction in heart rate (69.19%) and respiratory rate (40.70%) depending on the concentration of etomidate used during anesthetic induction. During the recovery period, there was cardiorespiratory reversibility to normality. Therefore, etomidate proved to be safe as an anesthetic agent for this species at concentrations of 2 to 3 mg.L ^-1^ for short-term anesthesia, but at higher doses the animals showed slow reversibility of anesthesia in a gradual manner and without excitability. The hemodynamic effect due to the rapid decrease in heart rate includes a negative factor of using higher concentrations of etomidate for *Colossome macropomum* anesthesia.

## Introduction

The growth of fishing and aquaculture activities worldwide is notable. In 2020, the movement of 214 million tons of aquatic animals and algae was recorded [1]. In Brazil, the production of farmed fish reached 860.355 tons in 2022 according to the Brazilian Aquaculture Association [2]. Considering the importance of aquaculture and animal welfare, the use of anesthetic agents in management practices such as biometrics and transportation is often necessary to avoid stress caused by these procedures and even to control pain in fish [3–9].

Anesthetic agents used in fish can be derived from plants or synthetic, and their effect can be monitored through biochemical, physiological, and behavioral aspects [10–14]. Among the most commonly used synthetic anesthetic agents are tricaine methanesulfonate (MS-222), benzocaine, etomidate, propofol, alfaxolone, among others, which can be used for sedation, anesthesia, and euthanasia [11, 15, 16]. The effects of anesthetic agents can vary depending on the species of fish studied [17]. Thus, it is necessary to research different anesthetic agents and their effects for their proper use in a particular species.

Etomidate is a non-barbiturate hypnotic agent based on imidazole that potentiates the activity of gamma-aminobutyric acid receptors and inhibits the release of neurotransmitters [18, 19]. Additionally, it causes an inhibitory effect on steroidogenesis, resulting in a decrease in cortisol levels and thus suppressing the stress response [20]. Etomidate has been investigated as an anesthetic agent in various fish species such as *Cyprinus carpio*, *Sander lucioperca*, *Perca fluviatilis*, *Sparus aurata* [21–24]. The study conducted by [23] used etomidate as an anesthetic agent in *Perca fluviatilis* L. and concluded from behavioral, hematological, and biochemical analysis that this agent was safe for inducing anesthesia in this species.

The tambaqui (*Colossoma macropomum*) plays an important role in Brazilian native fish farming, especially in the northern region [2, 25–27]. Some authors have investigated the effect of anesthetic agents in this species [28]. Evaluated the anesthetic effect of propofol and essential oil of *Nepeta cataria*, observing cardiac and ventilatory alterations. Considering the importance of this species and fish farming management, many other studies have used behavioral and electrophysiological methodologies to better understand the role these anesthetic agents play in tambaqui physiology, such as the study by [29–31]. Therefore, this study aims to investigate an appropriate concentration window for safe anesthesia in immersion baths with etomidate in the species *Colossoma macropomum*.

## Materials and methods

### Experimental animals

The individuals used were (n = 99) of the tambaqui species, *Colossoma macropomum*, acquired from the Laboratory of Aquaculture of Tropical Species (IFPA- Castanhal). The animals were stocked in aquariums at the Experimental Animal Facility of the Laboratory of Pharmacology and Toxicology of Natural Products at the Federal University of Pará (UFPA) in an environment with controlled temperature (25 to 28°C) and a photoperiod of 12 h light: 12 h dark. Feeding was conducted twice a day with commercial feed (32% protein) until satiety. Concurrently with siphoning to remove unconsumed food and feces, the water was partially renewed (approximately 20% of the tank volume) with water of the same origin. During the acclimatization period (10 days), water quality variables such as water temperature (°C), hydrogen potential (pH), and dissolved oxygen (DO) were monitored. Authorized by the Ethics Committee on Animal Use of the Federal University of Pará (CEUA/UFPA) under protocol number 7637241023.

### Acquisition of the medication

Etomidate (2 mg.ml^-1^) was acquired from Laboratório Midfarma, manufactured by Blau Farmacêutica S.A., located at Rua Adherbal Stresser, 84, São Paulo. The medication was diluted in water at concentrations of 2 mg.L ^-1^, 2.5 mg.L ^-1^, 3 mg.L ^-1^, 3.5 mg.L ^-1^ and 4 mg.L ^-1^. Subsequently, the animals were immersed in baths containing all these concentrations.

### Experimental design

#### Experiment with etomidate (ETM)

The tambaqui juveniles (27.38 ± 3.5g) were randomly distributed into the following treatments: a) control, b) fish subjected to immersion baths in ETM at a concentration of 2 mg.L ^-1^, c) 2.5 mg.L ^-1^, d) 3 mg.L ^-1^, e) 3.5 mg.L ^-1^ and f) 4 mg.L ^-1^. All fish were subjected to anesthetic induction by maintaining contact for a period of 10 minutes, and for anesthetic recovery, they were observed for 10 minutes in water without anesthetic. All experiments lasted for 10 minutes. For each recording, n = 9/treatment (immersion bath with ETM and recovery after immersion bath) was used, totaling 90 animals.

#### Experiment 1—Analysis of characteristic behavior of anesthetic induction and recovery

Considering the contact time with ETM concentrations, 2 mg.L ^-1^, 2.5 mg.L ^-1^, 3 mg.L ^-1^, 3.5 mg.L ^-1^ and 4 mg.L ^-1^ in immersion baths, the latency for the loss of posture reflex behavior was evaluated, characterized by lateral decubitus maintained for more than 15 seconds. Subsequently, the animals were removed from contact with ETM, and the latency for the recovery of posture reflex was evaluated, characterized by maintaining the posture for a period of 15 seconds. Additionally, the average latency for the loss of saccadic eye movement (during anesthetic induction) and the average latency for the recovery of saccadic eye movement (during anesthetic recovery) were assessed.

#### Experiment 2—Electrocardiogram Analysis (ECG)

For the analysis and monitoring of cardiac function, electrodes were made of 925 silver with a diameter of 0.3 mm and a length of 10 mm, which were later insulated with liquid insulation. They were made in a non-conjugated manner. The position used for fixing the reference electrode followed the indication of the cardiac vector (negative pole at the cardiac base and positive pole at the cardiac apex), and it was fixed in the ventral portion of the left opercular opening 0.2 mm after the end of the opercular cavity. The recording electrode was inserted 2.0 mm from the right opercular opening. The electrode captured the signal in lead D1. After that, the electrodes were connected to a high-impedance amplifier (Grass Technologies, Model P511) for electrocardiographic recordings. This allowed for the analysis of heart rate (bpm), amplitude of the QRS complex (mv), duration of the QRS complex (ms), RR intervals (ms), PQ intervals (ms), and ST intervals (ms).

#### Experiment 3—Recording of opercular activity during anesthetic induction and recovery

For the analysis of opercular activity, electrodes made of 925 silver with a diameter of 0.5 mm and a length of 15 mm were made. The electrodes were made in a conjugated manner at a distance of 5mm and insulated with liquid insulation. The position used for fixing the electrodes and recording the opercular beat was the central part of the right opercular opening. During the recording of the opercular beat, the frequency (beats per minute) and the power of the opercular beat (mV^2^/Hz) were evaluated.

### Recording and analysis of the records

The electrodes were connected to a digital data acquisition system through a high-impedance differential input amplifier (Grass Technologies, Model P511), adjusted with filtering of 0.3 and 300 Hz, with amplification of 2000X, and monitored with an oscilloscope (ProteK, Model 6510). The records were continuously digitized at a rate of 1 KHz on a computer equipped with a data acquisition board (National Instruments, Austin, TX), and were stored on a hard disk and subsequently processed using specialized software (LabVIEW express). The analysis of the acquired signals was possible with the aid of a tool built in the Python programming language version 2.7. The Numpy and Scipy libraries were used for mathematical processing, and the Matplotlib library was used for the graphical representation. The graphical interface was developed using the PyQt4 library [32]. The amplitude graphs demonstrate the potential differences between the reference and recording electrodes. The signal records were observed at 1000 samples per second.

### Statistical analysis

After verifying compliance with the assumptions of normality and variance homogeneity, through the Kolmogorov-Smirnov and Levene tests, respectively, comparisons of mean power values were made using one-way ANOVA, followed by Tukey’s test. GraphPad Prism® 8 software was used for the analyses, and a value of *p < 0.05, ** p < 0.01 and ***p < 0.001 were considered statistically significant in all cases.

## Results

### Etomidate caused loss of posture reflex and paralysis of eye movements at all tested concentrations

**Fig 1:**
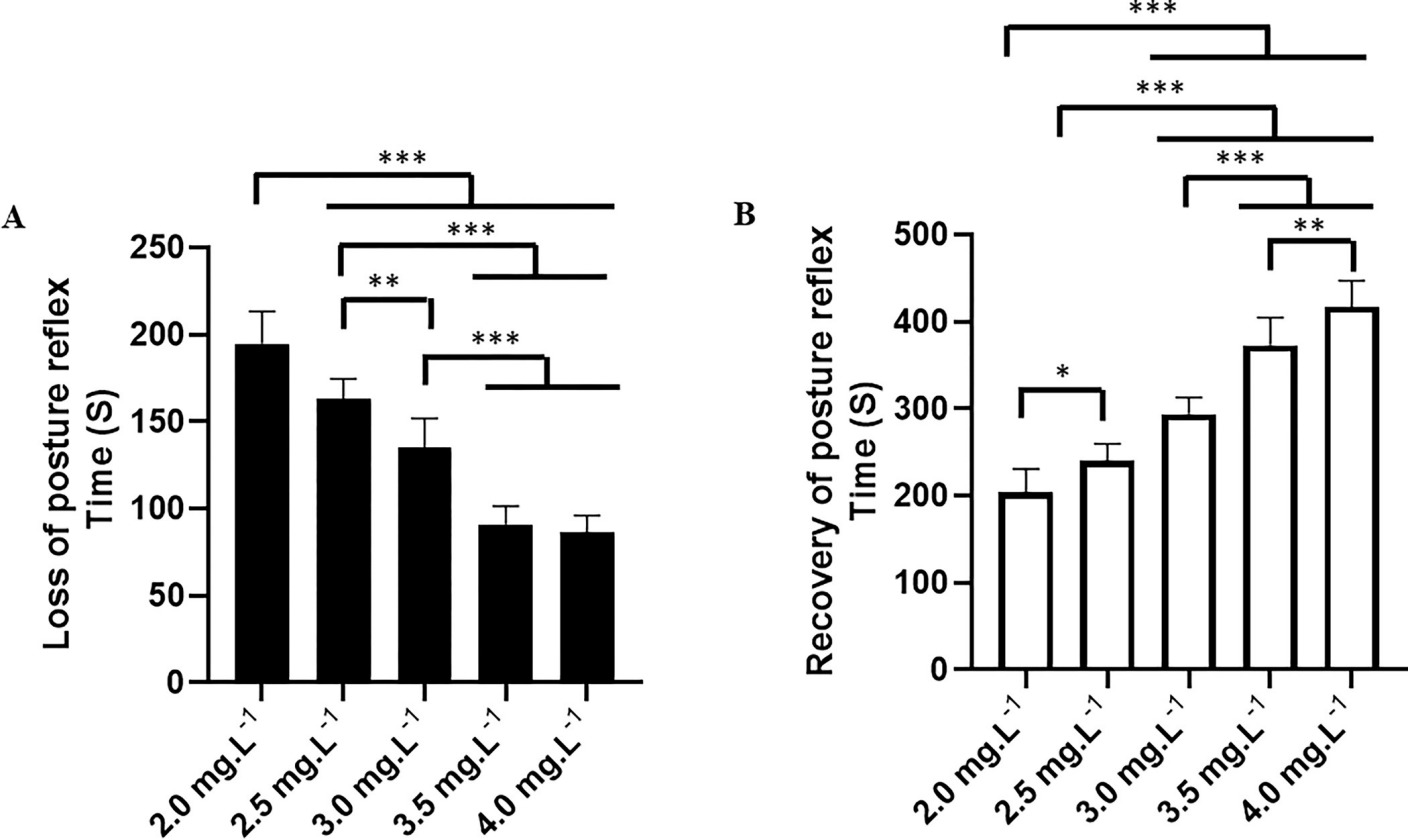
Mean latency of the postural reflex. Mean latencies for loss of the postural reflex during immersion baths with different concentrations of etomidate (A). Recovery of the postural reflex after contact with different concentrations of etomidate (B). (ANOVA followed by Tukey’s test; *p<0.05, **p<0.01, and ***p<0.001).

The behavioral analysis showed that etomidate caused a dose-dependent loss of the postural reflex (characterized by the inability to maintain an upright posture), with higher doses resulting in shorter latencies for the onset of posture reflex loss. Fish exposed to 2.0 mg.L^-1^ exhibited posture loss in 194.8 ± 18.54 s, while the group exposed to 2.5 mg.L^-1^ showed significantly shorter time with reflex loss in 163.1 ± 11.44 s. Fish treated with 3.0 mg.L^-1^ of etomidate had a mean loss of postural reflex of 135.0 ± 16.03 s, significantly faster than the groups receiving 2.0 mg.L^-1^ and 2.5 mg.L^-1^. For the group treated with 3.5 mg.L^-1^ (90.89 ± 10.48 s) and 4.0 mg.L^-1^ (86.11 ± 10.06 s), there was a difference compared to the other groups, but there was no difference between them (p = 0.949) (Fig 1A).

The recovery of the postural reflex in the group exposed to 2.0 mg.L^-1^ of etomidate occurred in 203.7 ± 26.78 s, which was shorter than the other groups: 2.5 mg.L^-1^ 240.0 ± 19.83 s), 3.0 mg.L^-1^ (294.0 ± 18.61 s), 3.5 mg.L^-1^ (373.2 ± 31.47 s), and 4.0 mg.L^-1^ (417.4 ± 30.03 s). All groups showed concentration-dependent recovery time, with higher concentrations requiring longer recovery times for the posture reflex, demonstrating slower reversibility of the effect for groups receiving higher concentrations (Fig 1B).

**Fig 2:**
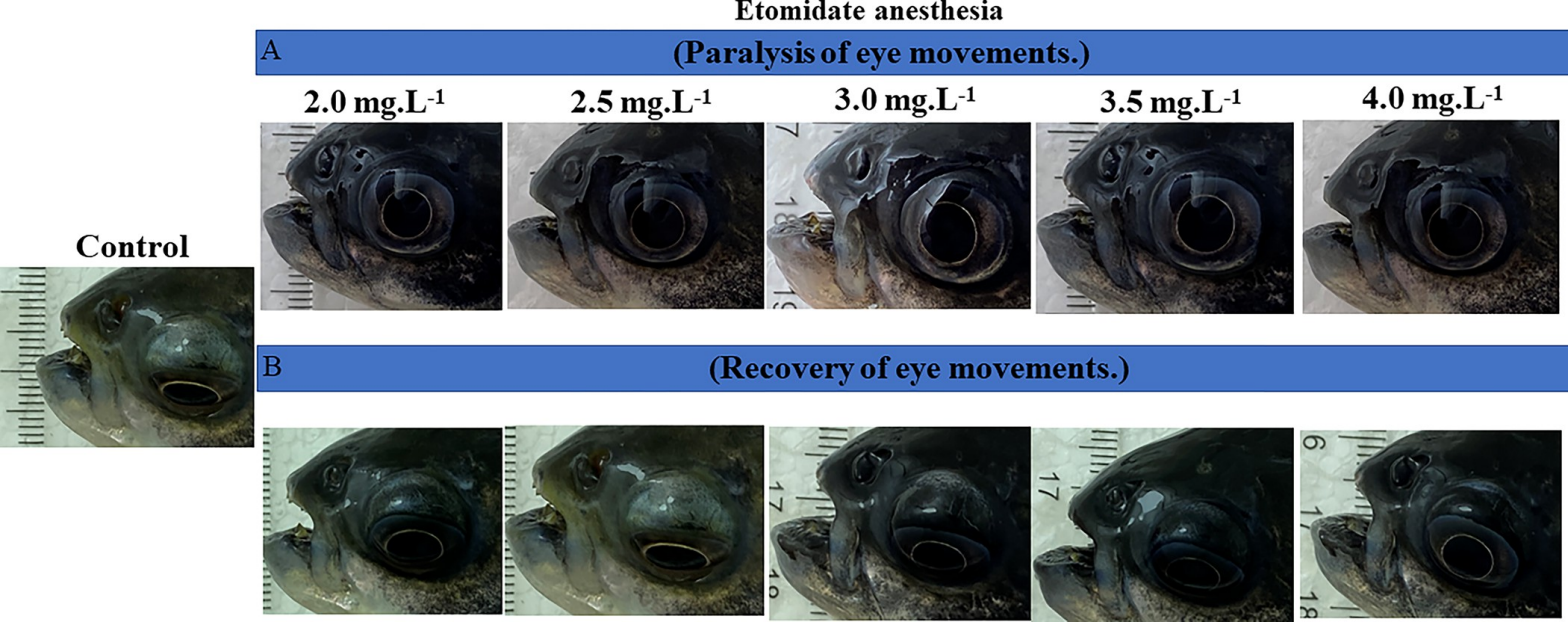
Presents a photographic record of tambaqui eye movement. Paralysis of eye movement occurs during treatment with different concentrations of ETM (A). Photographic record of tambaqui eye movement recovery in water free of etomidate (B). (Concentrations increase from left to right).

**Fig 3:**
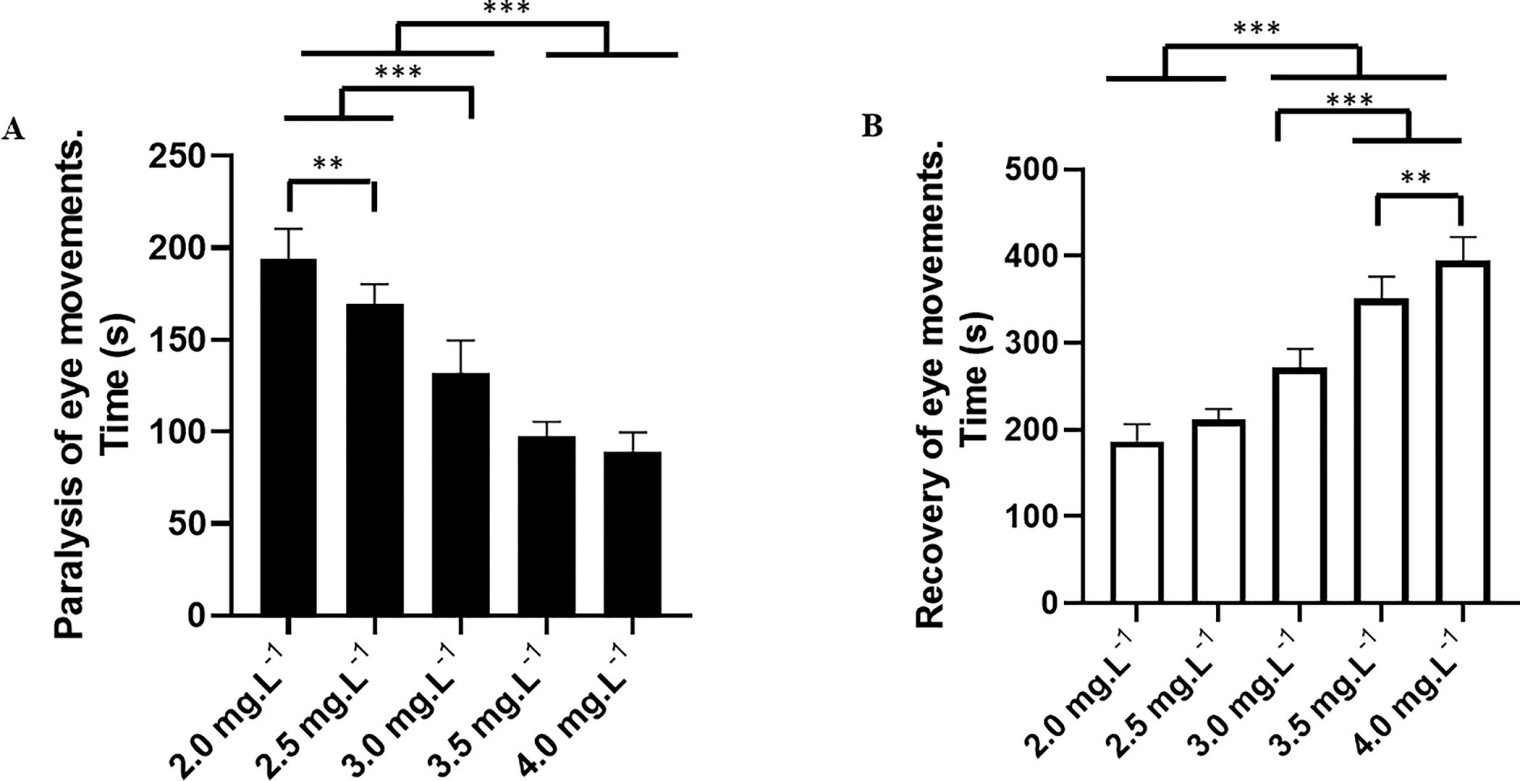
Mean latency of the saccadic movement. Mean latencies for loss of eye saccadic movement during immersion baths with different concentrations of etomidate (A). Recovery of eye saccadic movement after contact with different concentrations of etomidate (B). (ANOVA followed by Tukey’s test; **p<0.01 and ***p<0.001).

The behavioral analysis showed that etomidate (ETM) caused paralysis of the eye movements in tambaqui during anesthesia (Fig 2A), indicating deepening of anesthesia with ETM. The latency for paralysis of eye movement for the group treated with immersion bath containing 2 mg.L^-1^ of ETM had a mean latency of 193.7 ± 316.79 s, which was longer than the other groups: 2.5 mg.L^-1^ (169.8 ± 10.60 s), 3.0 mg.L^-1^ (131.8 ± 17.82 s), 3.5 mg.L^-1^ (97.67 ± 7.85 s), and 4 mg.L^-1^ (89.56 ± 10.19 s). Thus, all groups showed differences, as the latency for the loss of eye movements was shorter for the groups treated with higher concentrations (Fig 3A).

The recovery of eye movement after a 10-minute contact time in an ETM immersion bath (Fig 2B) for the group treated with 2.0 mg.L^-1^ had a mean of 186.6 ± 20.03 s, which was similar to the group treated with 2.5 mg.L^-1^ (211.7 ± 12.23 s) (p = 0.135). However, these times were shorter than the other treated groups: 3.0 mg.L^-1^ (271.4 ± 21.70 s), 3.5 mg.L^-1^ (350.7 ± 26.00 s), and 4.0 mg.L^-1^ (394.2 ± 27.59 s). The groups treated with higher concentrations had longer latencies for the recovery of eye movement (Fig 3B).

### Etomidate caused a concentration-dependent decrease in cardiac activity during treatment

**Fig 4:**
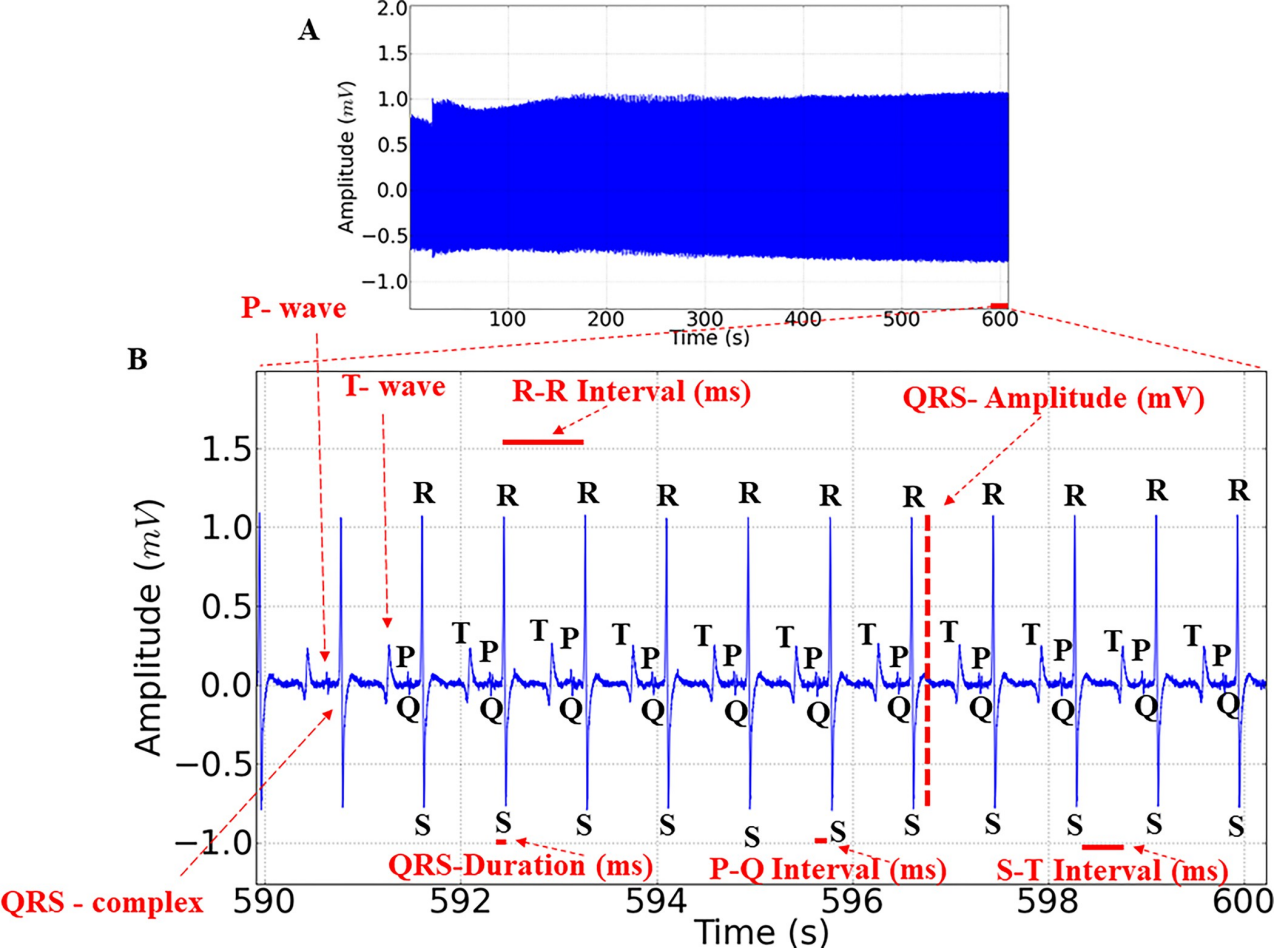
Graphic electrocardiogram. Electrocardiographic recording trace showing cardiac activity in juveniles of *Colossoma macopomum* (A), amplification of the last 10 seconds of the 10-minute recording (590 to 600s), demonstrating the graph elements evaluated in the study, including the P wave, QRS complex, and T wave, as well as variables such as heart rate (bpm), amplitude (mV), RR intervals, PQ intervals, QT intervals (ms), and duration of the QRS complex (ms), indicated in red (B).

Cardiac activity in the controls showed a mean frequency of 88.00 ± 2.44 bpm, with sinus rhythm and the presence of all cardiac deflections in the electrocardiogram (Fig 4A). In a 10-second amplification, all cardiac waves and complexes could be observed, including the P wave, QRS complex, and T wave, enabling the evaluation of intervals during ETM immersion and its recovery. Thus, we could identify atrial activity represented by P waves, ventricular activity by QRS complexes and ventricular repolarization by T waves (Fig 4B). The graph elements that allowed the data evaluation are indicated in Fig 4B.

**Fig 5:**
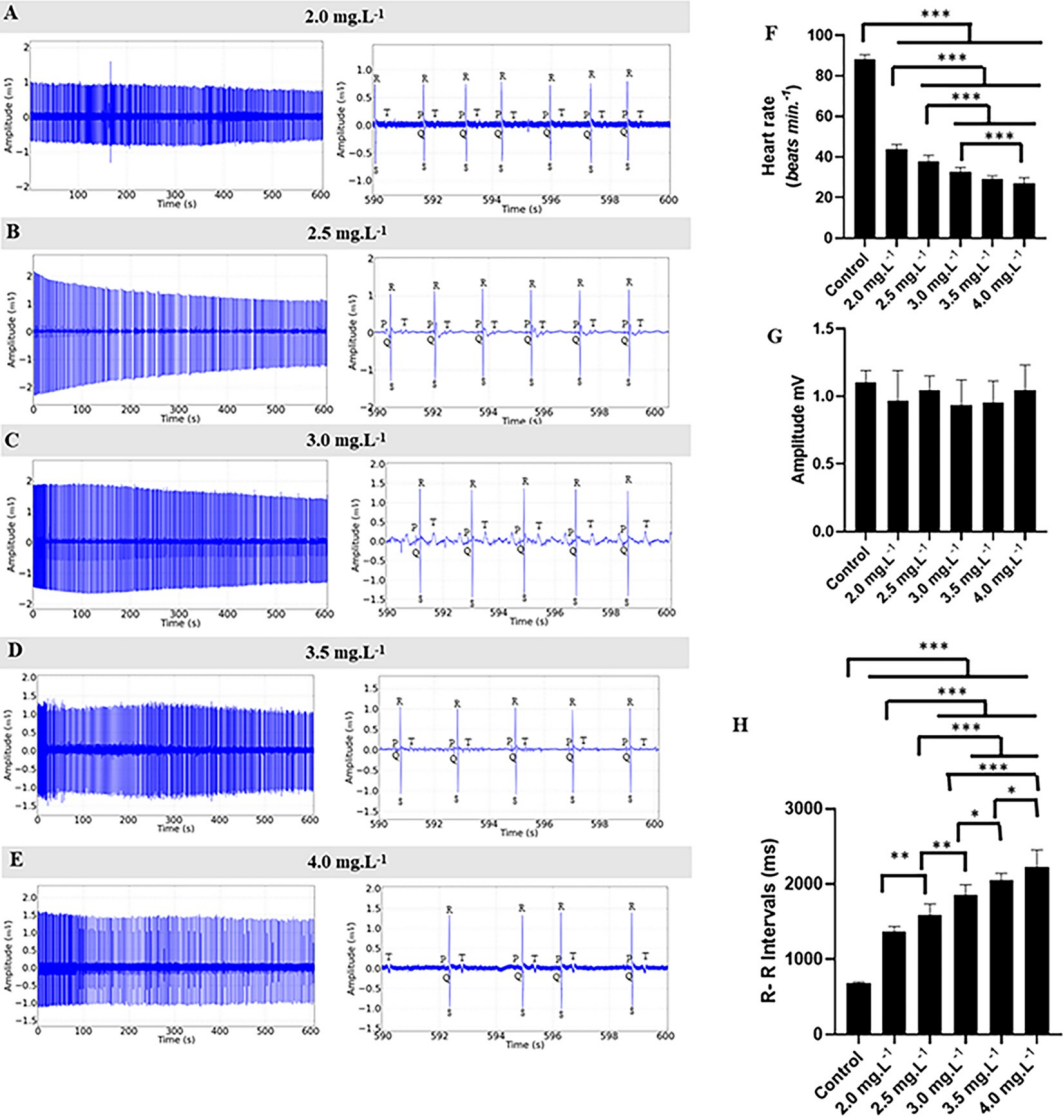
Electrocardiogram during the treatment. Cardiac activity in juvenile *Colossoma macropomum* during immersion baths with different concentrations of etomidate (left). Amplification of the record in the last 10 seconds (590–600 s) (center) to identify cardiac deflections for treatments at concentrations of 2.0 mg.L^-1^ (A), 2.5 mg.L^-1^ (B), 3.0 mg.L^-1^ (C), 3.5 mg.L^-1^ (D), and 4.0 mg.L^-1^ (E). Mean values of heart rate in beats per minute (bpm) (F), mean values of QRS complex amplitude (mV) (G), mean values of RR intervals (ms) (H) during ETM treatment. (ANOVA followed by Tukey’s test; *P<0.05, **p<0.01, ***p<0.001; n = 9).

During treatment with ETM at concentrations of 2.0 mg.L^-1^, 2.5 mg.L^-1^, 3.0 mg.L^-1^, 3.5 mg.L^-1^ and 4.0 mg.L^-1^, the ECG showed a concentration-dependent decrease in cardiac excitability (Fig 5A–5E). However, at the highest concentration (4.0 mg.L^-1^), a marked decrease in cardiac activity was observed within the 10-minute observation period. Nevertheless, the rhythm remained sinusoidal at all tested concentrations (Fig 5). The heart rate decreased by 50.25% in the group exposed to 2.0 mg.L^-1^ and by 57.06% in the fish exposed to 2.5 mg.L^-1^, compared to the control. Fish that received 3.0 mg.L^-1^, 3.5 mg.L^-1^, and 4.0 mg.L^-1^ exhibited more pronounced bradycardia compared to the control, with respective decreases in cardiac function of (63.13%, 66.67%, and 69.19%) (Fig 5).

The heart rate was significantly affected by increasing concentrations of ETM. Controls had a mean of 88.00 ± 2.44 bpm, which was higher than the treated groups. The group treated with 2.0 mg.L^-1^ had a mean of (43.78 ± 2.33 bpm), the group treated with 2.5 mg.L^-1^ (37.78 ± 3.073 bpm), the group treated with 3.0 mg.L^-1^ (32.44 ± 2.40 bpm), the group treated with 3.5 mg.L^-1^ (29.33 ± 1.41 bpm), and the group treated with 4.0 mg.L^-1^ (27.11 ± 2.66 bpm). All groups showed statistical differences from each other except for the 3.0 mg.L^-1^ and 3.5 mg.L^-1^ groups (p = 0.093) and the 3.5 mg.L^-1^ and 4.0 mg.L^-1^ groups (p = 0.396) (Fig 5F).

The mean amplitude of the QRS complex in the control group was 1.103 ± 0.088 mV, which was similar to the other groups. The group treated with 2.0 mg.L^-1^ had a mean amplitude of 0.967 ± 0.222 mV, the group exposed to 2.5 mg.L^-1^ (1.048 ± 0.102 mV), the group with 3.0 mg.L^-1^ of ETM (0.933 ± 0.188 mV), the group with 3.5 mg.L^-1^ (0.954 ± 0.158 mV), and the group with 4.0 mg.L^-1^ (1.043 ± 0.188 mV) showed no statistical differences between them (F(5,48) = 1.438; p = 0.227) (Fig 5G).

The mean RR interval in the control group was 681.7 ± 19.00 ms, which was lower compared to the other groups. The group treated with 2.0 mg.L^-1^ had a mean RR interval of 1368.0 ± 67.78 ms, which differed from the mean of the fish exposed to 2.5 mg.L^-1^ (1593.0 ± 139.9ms), the 3.0 mg.L^-1^ group (1854.0 ± 136.4 ms), the 3.5 mg.L^-1^ group (2048.0 ± 95.52 ms), and the 4.0 mg.L^-1^ group (2231.0 ± 223,2 ms), all of which showed statistical differences between them (Fig 5H).

**Fig 6:**
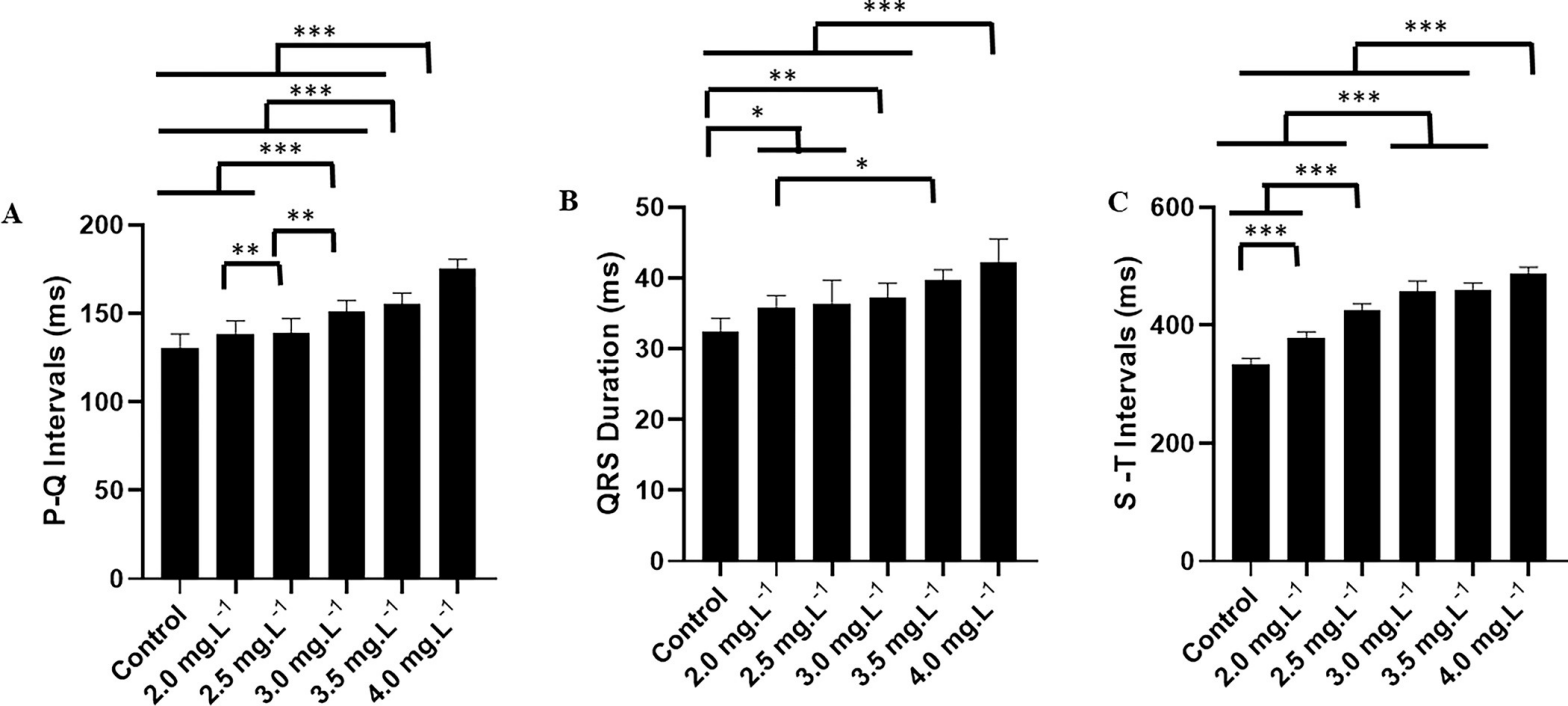
Mean of the electrocardiographic intervals during the treatment. Mean values of PQ interval (ms) (A), duration of QRS complex (ms) (B), and ST interval (ms) (C) during exposure to ETM at 2.0 mg.L^-1^, 2.5 mg.L^-1^, 3.0 mg.L^-1^, 3.5 mg.L^-1^, and 4.0 mg.L^-1^. (ANOVA followed by Tukey’s test; *P<0.05, **p<0.01, ***p<0.001; n = 9).

The mean PQ interval for the control group was 130.6 ± 7.92 ms, which did not differ from the groups at 2.0 mg.L^-1^ (138.6 ± 7.41 ms) (p = 0.158) and 2.5 mg.L^-1^ (139.2 ± 7.87 ms) (p = 0.102). However, it was shorter than the means of the groups treated with 3.0 mg.L^-1^ (151.3 ± 6.124 ms), 3.5 mg.L^-1^ (155.1 ± 6.412 ms), and 4.0 mg.L^-1^ (175.2 ± 5.31 ms). The groups treated with 3.0 mg.L^-1^ and 3.5 mg.L^-1^ were similar (p = 0.853) (Fig 6A).

The average duration of the QRS complex for the control group during induction was 32.44 ± 1.87 ms, which was shorter than the other groups. For the groups at 2.0 mg.L^-1^ (35.89 ± 1.61 ms) and 2.5 mg.L^-1^ (36.44 ± 3.38 ms) (p = 0.996), and 3.0 mg.L^-1^ (37.22 ± 2.048 ms) (p = 0.842), they were similar. The 4.0 mg.L^-1^ group (42.22 ± 3.308 ms) was larger than all groups. The 3.5 mg.L^-1^ group (39.67 ± 1.50 ms) was larger than the 2.0 mg.L^-1^ treated group (Fig 6B).

For the control group, the mean ST interval during induction was 333.3 ± 10.76 ms, which was lower than the other groups: the group treated with 2.0 mg.L^-1^ (378.6 ± 10.26 ms), 2.5 mg.L^-1^ (425.4 ± 11.49 ms), 3.0 mg.L^-1^ (456.4 ± 18.37 ms), 3.5 mg.L^-1^ (460.8 ± 11.13 ms), and 4.0 mg.L^-1^ (487.9 ± 10.87 ms). All groups showed statistical differences between each other, however, the 3.0 mg.L^-1^ and 3.5 mg.L^-1^ groups were similar (p = 0.981) (Fig 6C).

**Fig 7:**
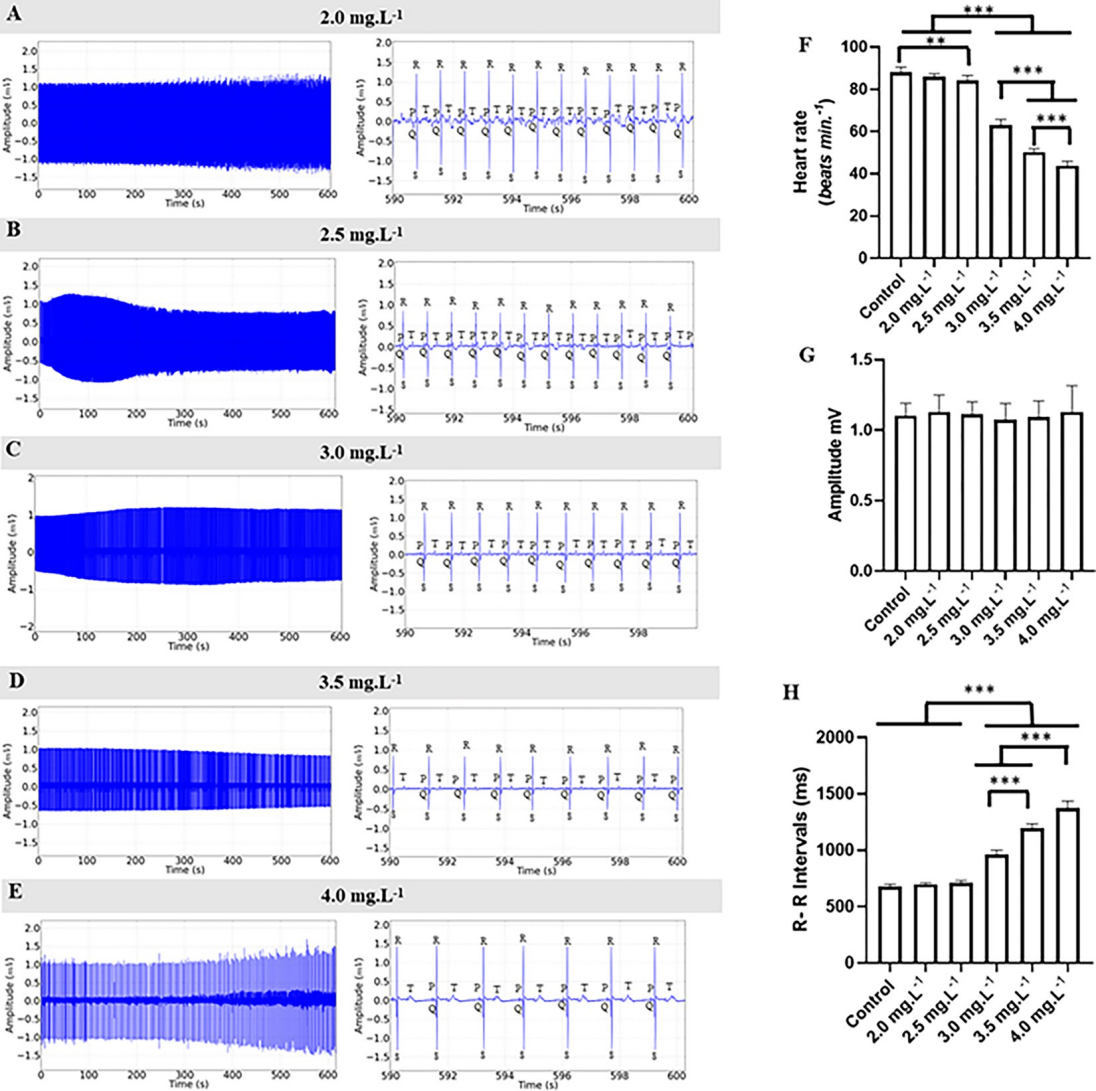
Electrocardiogram during the recovery. Cardiac activity in *Colossoma macropomum* juveniles during recovery after immersion baths with different concentrations of ETM (left). Amplification of the record in the last 10 seconds (590–600 s) for identification of cardiac deflections (center), during the recovery period after immersion baths with the following ETM concentrations: 2.0 mg.L^-1^ (A), 2.5 mg.L^-1^ (B), 3.0 mg.L^-1^ (C), 3.5 mg.L^-1^ (D), and 4.0 mg.L^-1^ (E). Mean values of heart rate (bpm) (F); mean values of QRS complex amplitude (mV) (G); mean RR intervals (ms) (H). (ANOVA followed by Tukey’s test; *P<0.05, **p<0.01, ***p<0.001; n = 9).

During the recovery phase from exposure to ETM at the concentrations used 2.0 mg.L^-1^, 2.5 mg.L^-1^, 3.0 mg.L^-1^, 3.5 mg.L^-1^, and 4.0 mg.L^-1^, reversibility of the electrocardiographic changes was observed, albeit slowly (Fig 7A–7E). The cardiac rhythm was sinusoidal, with a gradual increase in heart rate approaching that of the control group. However, at higher concentrations, reversibility took longer than 10 minutes. Thus, for the group treated with 2.0 mg.L^-1^ of ETM, the heart rate was 97.47% of the control (Fig 7A). For the other groups, 2.5 mg.L^-1^ (95.70%) (Fig 7B), 3.0 mg.L^-1^ (71.46%) (Fig 7C), 3.5 mg.L^-1^ (57.06%) (Fig 7D), and 4.0 mg.L^-1^ (49.75%) (Fig 7E), heart rate values were observed. No arrhythmias were observed during the recovery period, and the reversal of ETM’s effects on the heart was gradual and dependent on the concentration used in the treatment.

During the recovery period, the control group had a mean heart rate of 88.00 ± 2.44 bpm, which was not statistically different from the 2.0 mg.L^-1^ group with a mean of 85.78 ± 1.56 bpm (p = 0.280), but differed significantly from the other groups. The 2.5 mg.L^-1^ group (84.22 ± 2.33 bpm), 3.0 mg.L^-1^ group (62.89 ± 2.84 bpm), 3.5 mg.L^-1^ group (50.22 ± 1.56 bpm), and 4.0 mg.L^-1^ group (43.78 ± 2.10 bpm) all differed significantly from the control. The groups treated with 2.0 mg.L^-1^ and 2.5 mg.L^-1^ had higher heart rates compared to those treated with higher concentrations. The 3.0 mg.L^-1^ group had a higher heart rate than the 3.5 mg.L^-1^ and 4.0 mg.L^-1^ groups. The 4.0 mg.L^-1^ group had the lowest mean heart rate during the recovery period, however, the rhythm was sinusoidal with a gradual return to normal, confirming that higher concentrations required more time for the reversal of bradycardia (Fig 7F).

The amplitude of the QRS complex during recovery for the control group was 1.103 ± 0.088 mV. The groups treated with 2.0 mg.L^-1^ (1.128 ± 0.122 mV), 2.5 mg.L^-1^ (1.12 ± 0.088 mV), 3.0 mg.L^-1^ (1.074 ± 0.115 mV), 3.5 mg.L^-1^ (1.09 ± 0.11 mV), and 4.0 mg.L^-1^ (1.131 ± 0.184 mV) were all similar (F(5, 48) = 0.2785, p = 0.922) (Fig 7G).

The average RR interval during recovery for the control group was 681.7 ± 19.00 ms, which did not differ statistically from the 2.0 mg.L^-1^ group (699.1 ± 12.94 ms) (p = 0.916) and the 2.5 mg.L^-1^ group (712.4 ± 20.09 ms) (p = 0.501). However, these groups were lower than the other groups. The 3.0 mg.L^-1^ group (958.8 ± 42.05 ms) was lower than the 3.5 mg.L^-1^ (1194 ± 39.09 ms) and 4.0 mg.L^-1^ (1373 ± 63.53 ms) groups (Fig 7H).

**Fig 8:**
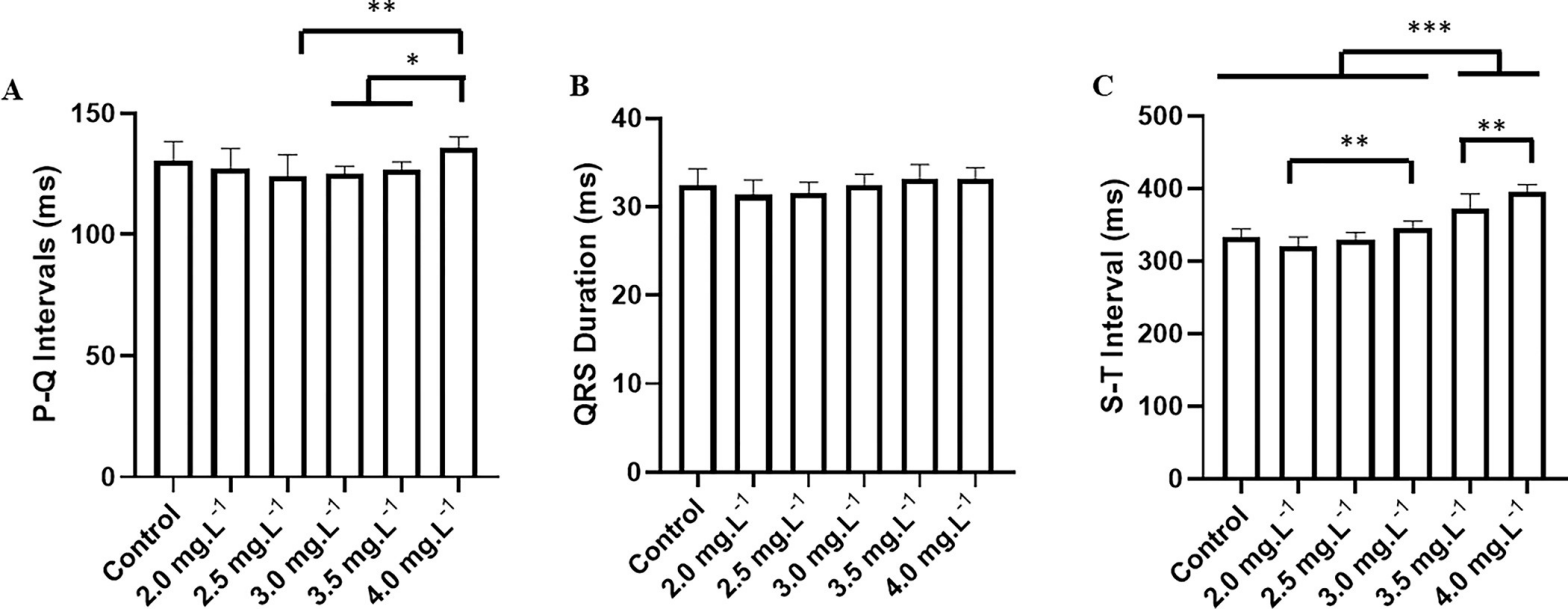
Mean of the electrocardiographic intervals during the recovery. Mean values of cardiac parameters during recovery from exposure to different concentrations of ETM in immersion bath at 2.0 mg.L^-1^, 2.5 mg.L^-1^, 3.0 mg.L^-1^, 3.5 mg.L^-1^ and 4.0 mg.L^-1^. Mean values of PQ intervals (ms) (A); duration of the QRS complex (ms) (B) and QT interval (ms) (C). (ANOVA followed by Tukey’s test; *P<0.05, **p<0.01, ***p<0.001; n = 9).

The PQ interval during recovery in the control group was 130.6 ± 7.92 ms, which did not differ from the other groups: 2.0 mg.L^-1^ (127.3 ± 8.10 ms) (p = 0.893), 2.5 mg.L^-1^ (124.2 ± 8.715 ms) (p = 0.310), 3.0 mg.L^-1^ (125.0 ± 3.16 ms) (p = 0.455), 3.5 mg.L^-1^ (126.6 ± 3.43 ms) (p = 0.773), and 4.0 mg.L^-1^ (135.7 ± 4.69 ms) (p = 0.547). The group treated with 4.0 mg.L^-1^ was higher than the groups 2.0 mg.L^-1^, 2.5 mg.L^-1^, 3.0 mg.L^-1^, and 3.5 mg.L^-1^ (Fig 8A).

The duration of the QRS complex during recovery did not differ between groups: the control group (32.44 ± 1.87 ms) and fish treated with 2.0 mg.L^-1^ (31.33 ± 1.73 ms), 2.5 mg.L^-1^ (31.56 ± 32.44 ms), 3.0 mg.L^-1^ (32.44 ± 1.23 ms), 3.5 mg.L^-1^ (33.22 ± 1.56 ms), and 4.0 mg.L^-1^ (33.22 ± 1.20 ms) (F(5, 48) = 2.567; p = 0.039) (Fig 8B).

During recovery, the ST interval for the control was 333.3 ± 10.76 ms, similar to that of fish exposed to 2.0 mg.L^-1^ (320.4 ± 12.62 ms) (p = 0.262), 2.5 mg.L^-1^ (329.9 ± 9.68 ms) (p = 0.990), 3.0 mg.L^-1^ (344.9 ± 9.714 ms) (p = 0.379). However, it was shorter than the groups 3.5 mg.L^-1^ (372.6 ± 19.75 ms) and 4.0 mg.L^-1^ (395.9 ± 9.18 ms). All groups were shorter than the group treated with 4.0 mg.L^-1^ (Fig 8C).

**Fig 9:**
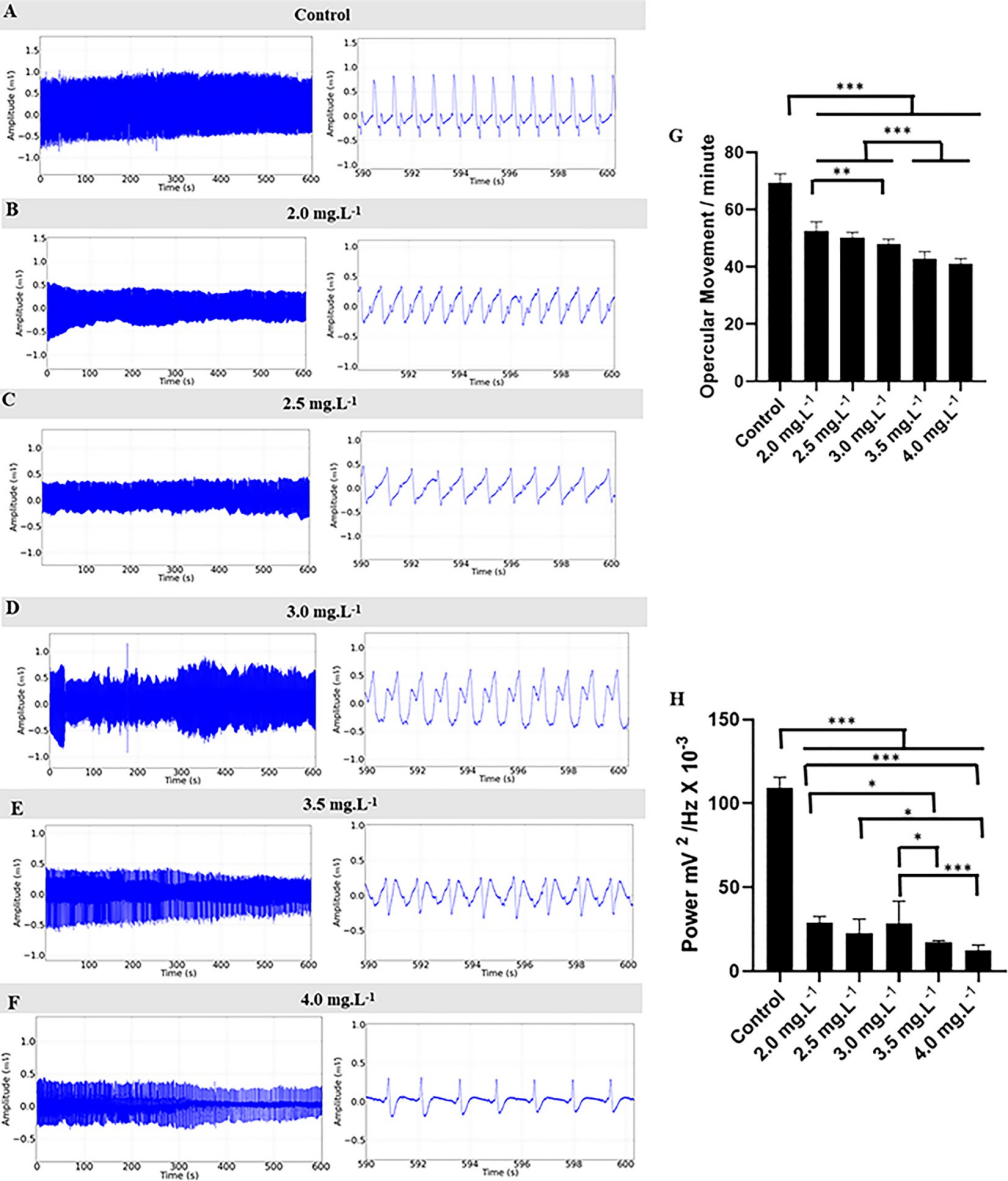
Opercular activity during the treatment. Record of opercular activity in juveniles of *Colossomo macropomum* during immersion bath in different concentrations of ETM (left). Amplification of the record in the last 10 seconds (590–600 s) (center) for identification of changes in opercular movement per minute (omm), for the control group (A), for treatment at concentrations of 2.0 mg.L^-1^ (B), 2.5 mg.L^-1^ (C), 3.0 mg.L^-1^ (D), 3.5 mg.L^-1^ (E) and 4.0 mg.L^-1^ (F). Mean values of opercular movement frequency per minute (G) and mean power of opercular movement (mV^2^/Hz X 10^−3^) (H). (ANOVA followed by Tukey’s test; *P<0.05, **p<0.01, ***p<0.001; n = 9).

During treatment with ETM at concentrations of 2.0 mg.L^-1^, 2.5 mg.L^-1^, 3.0 mg.L^-1^, 3.5 mg.L^-1^ and 4.0 mg.L^-1^, the opercular movement frequency (omm) showed concentration-dependent decrease (Fig 9A–9F). The control group had a mean of 69.33 ± 3.162 omm and was higher than the other groups: 2.0 mg.L^-1^ with a mean of 52.56 ± 3.208 omm and a decrease of 24.18%; 2.5 mg.L^-1^ (50.22 ± 1.85 omm) (27.56%), 3.0 mg.L^-1^ (48.00 ± 1.73 omm) (30.73%), 3.5 mg.L^-1^ (42.67 ± 2.64 omm) (38.45%), and 4.0 mg.L^-1^ (41.11 ± 1.76 omm) (40.70%). The groups treated with 3.5 mg.L^-1^ and 4.0 mg.L^-1^ were similar (p = 0.766), but lower than the other groups. The groups treated with 2.0 mg.L^-1^ and 2.5 mg.L^-1^ were similar (p = 0.358), as were the groups 2.5 mg.L^-1^ and 3.0 mg.L^-1^ (p = 0.413) (Fig 9G).

A decrease in power in the opercular movement (omm) recordings was observed during anesthetic induction. The control group had a mean power of 109.0 ± 6.515 mV^2^ /Hz X 10^−3^, which was higher than all the power means of the groups treated with 2.0 mg.L^-1^ (29.08 ± 3.56 mV^2^/Hz X 10^−3^), 2.5 mg.L^-1^ (22.78 ± 8.42 mV^2^/Hz X 10^−3^), 3.0 mg.L^-1^ (28.42 ± 13.38 mV^2^/Hz X 10^−3^), 3.5 mg.L^-1^ (17.29 ± 0.99 mV^2^/Hz X 10^−3^), and 4.0 mg.L^-1^ (12.40 ± 3.24 mV^2^/Hz X 10^−3^). Animals treated with 3.5 mg.L^-1^ and 4.0 mg.L^-1^ were similar (p = 0.750) (Fig 9H).

**Fig 10:**
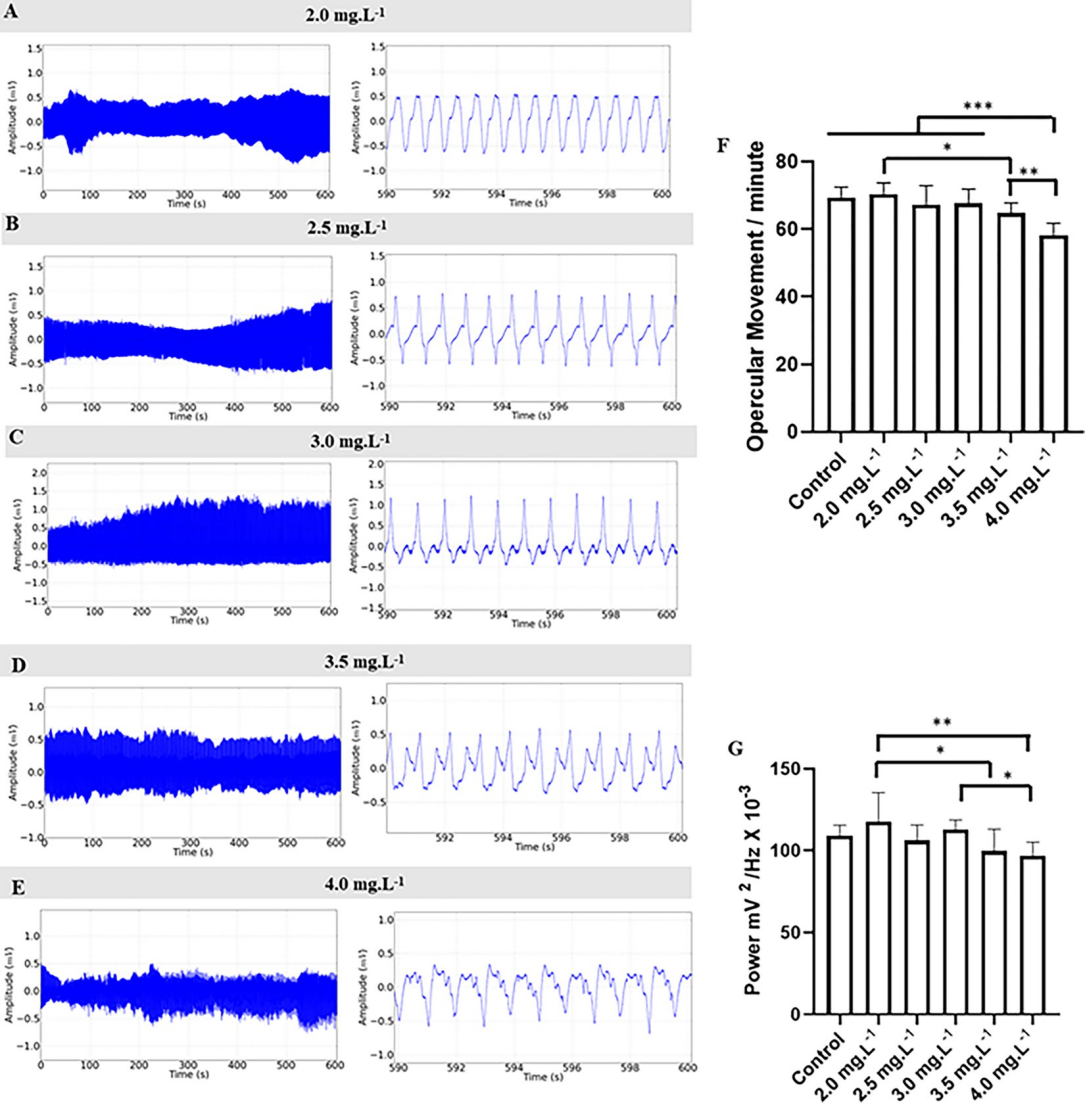
Opercular activity during the recovery. Opercular movement recording in juveniles of *Colossomo macropomum*, during recovery after immersion bath with different concentrations of ETM (left). Amplification of the recording in the last 10 seconds (590–600 s) for identification of the records (center), in the recovery period after immersion bath with the following concentrations of ETM: 2.0 mg.L^-1^ (A), 2.5 mg.L^-1^ (B), 3.0 mg.L^-1^ (C), 3.5 mg.L^-1^ (D), and 4.0 mg.L^-1^ (E). Mean values of opercular movement per minute (F); mean power values of opercular movement (mV^2^/Hz X 10^−3^) (G). (ANOVA followed by Tukey’s test; *P<0.05, **p<0.01, ***p<0.001; n = 9).

During the anesthesia recovery period, the opercular movement frequency (omm) showed concentration-dependent recovery (Fig 10A–10E). The control group had a mean of 69.33 ± 3.162 omm and was similar to the groups: 2.0 mg.L^-1^ with a mean of 70.44 ± 3.28 omm (p = 0.989); 2.5 mg.L^-1^ (67.33 ± 5.56 omm) (p = 0.877), 3.0 mg.L^-1^ (67.78 ± 4.055 omm) (p = 0.954), 3.5 mg.L^-1^ (64.89 ± 2.84 omm) (p = 0.159). The group treated with 4.0 mg.L^-1^ (58.22 ± 3.52 omm) was lower compared to the other groups (Fig 10F).

The increase in power in opercular movement records (omm) was observed during anesthesia recovery. The control group had a mean power of 109.0 ± 6.515 mV2/Hz X 10–3, which was similar to the recovery groups of the following treatments: 2.0 mg.L^-1^ (117.6 ± 17.93 mV^2^/Hz X 10^−3^) (p = 0.580), 2.5 mg.L^-1^ (106.2 ± 9.41 mV^2^/Hz X 10^−3^) (p = 0.994), 3.0 mg.L^-1^ (112.8 ± 5.94 mV^2^/Hz X 10^−3^) (p = 0.977), 3.5 mg.L^-1^ (99.78 ± 13.36 mV^2^/Hz X 10^−3^) (p = 0.496) and 4.0 mg.L^-1^ (96.78 ± 8.48 mV^2^/Hz X 10^−3^) (p = 0.198). The group treated with 4.0 mg.L^-1^ was lower than the groups treated with 2.0 mg.L^-1^ and 3.0 mg.L^-1^ (Fig 10G).

## Discussion

Although there are several studies in the literature using etomidate as an anesthetic agent and for research in fish neurosciences [18, 33–35], this is the first time it has been tested in the species *Colossoma macropomum* to establish safe concentrations for anesthesia. In the present study, all etomidate concentrations provided anesthesia induction in tambaqui with a concentration-dependent profile. Thus, at a concentration of 2.0 mg.L^-1^, the induction time was slightly longer than 3 minutes, while all other tested concentrations allowed induction in less than 3 minutes, which is considered an ideal induction time for anesthesia in several species and developmental stages [17, 36–38]. Studies conducted at a concentration of 2.0 mg.L^-1^ of etomidate showed that the time required for anesthesia induction in *Perca fluviatilis* L. and *Sparus aurata* L. was approximately 2 and 4 minutes, respectively [23, 24]. Accordingly, the investigation of anesthetic agents and individual organism responses reinforces the need for knowledge of safe concentration ranges for each species.

When evaluating the recovery time of the posture reflex, all concentrations allowed recovery in less than 7 minutes. In the case of concentrations of 2–3 mg.L^-1^, the recovery of the posture reflex occurred in less than 5 minutes, a period proposed in previous studies [17]. While concentrations of 3.5 and 4 mg.L^-1^ caused a delay in the recovery of the posture reflex, no complications were observed during this process. In the study conducted by [39] with *Xiphophotus maculatu*, the recovery time of the posture reflex ranged from 13–23 minutes. Therefore, anesthetic recovery with etomidate in tambaqui, even at higher concentrations, approached the time proposed in the literature and was faster than anesthetic recovery using some natural compounds [40–44].

Saccadic eye movements occur somatically, thus there is a relationship between the presence of eye movement and the maintenance of posture reflex, as well as the depth of anesthesia. So general anesthetic agents should exhibit myorelaxation as a characteristic. However, some substances used as anesthetics have difficulty in suppressing this type of behavior, such as lidocaine [45, 46].

The analyses demonstrated that the loss of eye movement occurred near the loss of posture reflex, enabling the evaluation of the anesthetic effect from a behavioral perspective. This finding was also similar to that found by [47], where through cryoanesthesia in tambaquis, it was observed that as the anesthesia deepened, the animal also lost saccadic eye movement, which was completely reversible upon anesthesia recovery. The work by [45] indicated that eye movements cease due to a lack of behavioral impulse. The results obtained from the time of loss of posture reflex and eye movement reveal their nearly simultaneous loss. In contrast, the recovery of eye movement preceded the recovery of posture reflex, demonstrating that this reflexive behavior activates more rapidly.

In relation to the cardiographic analyses, the anesthesia induction process caused a decrease in heart rate with characteristics of sinus bradycardia, which was more intense at higher concentrations. Consequently, due to the decline in heart rate, an increase in the RR interval was observed. The decrease in heart rate was also recorded in the study by [48], using tricaine methanesulfonate (MS-222) as an anesthetic agent in tambaqui. Therefore, cardiac depression caused by anesthetic agents in fish may be an indirect result of central nervous system depression [28, 31, 48, 49].

Despite the cardiac depression observed at all concentrations, there was reversibility of cardiac effects to normality during the recovery period, and more slowly at higher concentrations. Even though total heart rate did not recover at higher concentrations (3–4 mg.L^-1^), no arrhythmias occurred and the rhythm remained sinusoidal. During recovery, delayed ventricular depolarization was not detected, however, the ST interval remained prolonged at concentrations of 3.5 and 4 mg.L^-1^. In the study by [28], an arrhythmia pattern was observed during anesthetic recovery using propofol in tambaqui, which was not observed in this study. Furthermore, the use of tricaine by [48] also presented arrhythmia in tambaqui during recovery. Therefore, etomidate did not demonstrate arrhythmia patterns when compared to other synthetic anesthetic agents applied in tambaqui.

Another important aspect during anesthesia is respiratory activity. Respiratory rhythm can be abruptly affected by anesthetic agents, so it is crucial to monitor respiratory response during anesthesia. When subjected to anesthesia, respiratory rate is reduced through suppression of the gill apparatus but should still remain regular [50–52]. Thus, monitoring of respiratory frequency revealed its decrease, ranging between 24–40%, depending on the dose administered. In despite of that, the respiratory rate of tambaqui subjected to etomidate was higher compared to exposure of this species to propofol and menthol [28, 30]. Additionally, respiratory power was also evaluated to analyze respiratory quality. In the finding of [53], using citronella essential oil as an anesthetic in tambaqui, it was possible to observe that both power and respiratory frequency decreased to levels lower than those found in the present study. The decrease in both opercular frequency and power at higher concentrations of etomidate may affect oxygen diffusion in the secondary gill filament, which may be related to the delay in the recovery of the postural reflex in a dose-dependent manner.

Even with the decrease in respiratory quality, fish exposed to etomidate were able to restore respiratory activity during recovery. Thus, during the recovery period, the respiratory frequency of the tested concentrations was similar to the control, except at the concentration of 4 mg.L^-1^, which was 15% below the control. At this concentration, our result was similar to menthol and citronella during recovery [31, 53]. Furthermore, respiratory power was restored in all tested concentrations, being lower at 4 mg.L^-1^. Therefore, despite the higher concentration being slightly below the control value when compared, all tested concentrations had a reversible effect on respiration during recovery.

In conclusion, our study allowed us to assess the reversibility of behavioral and cardiorespiratory effects in tambaqui, using different concentrations of etomidate as a safe anesthetic agent for this species within the concentration range of 2 to 3 mg.L^-1^. Anesthesia with higher concentrations of etomidate in immersion baths may lead to hemodynamic changes due to cardiac and respiratory compromise in *Colossoma macropomum*.

## Supporting information

S1 File All data from our work is available in the ZIP file found in the supporting information. (ZIP)
